# Supplementary material for: Construction of room temperature phosphorescent materials with ultralong lifetime by in-situ derivation strategy
Source: Nat Commun. 2023 Jul 13;14:4164. doi: 10.1038/s41467-023-39795-6 (PMC10344959; doi:10.1038/s41467-023-39795-6)
Supplement: Supplementary file 1 — Supplementary Information [file 41467_2023_39795_MOESM1_ESM.pdf]

Supplementary Information

Construction of room temperature phosphorescent materials with ultralong lifetime by in-situ derivation strategy

Qinglong Jia[1,+], Xilong Yan[1,2,3,4,+], Bowei Wang[1,2,3,4,*], Jiayi Li[1], Wensheng Xu[1], Zhuoyao Shen[1], Changchang Bo[1], Yang Li[1,3,4], Ligong Chen,[1,2,3,4,*]

[1] School of Chemical Engineering and Technology, Tianjin University, Tianjin 300350, P. R. China.

[2] Zhejiang Institute of Tianjin University, Shaoxing, 312300, P.R. China.

[3] Tianjin Engineering Research Center of Functional Fine Chemicals, Tianjin 300350, P.R. China.

[4] Guangdong Laboratory of Chemistry and Fine Chemical Industry Jieyang Center, Guangdong Province 522000, P.R. China

[+] These authors contributed equally: Qinglong Jia, Xilong Yan.

* Corresponding author at: School of Chemical Engineering and Technology, Tianjin University, Tianjin 300350, P. R. China.

E-mail: bwwang@tju.edu.cn (Bowei Wang, ORCID ID: 0000-0002-9400-0698); lgchen@tju.edu.cn (Ligong Chen, ORCID ID: 0000-0002-3442-5694).

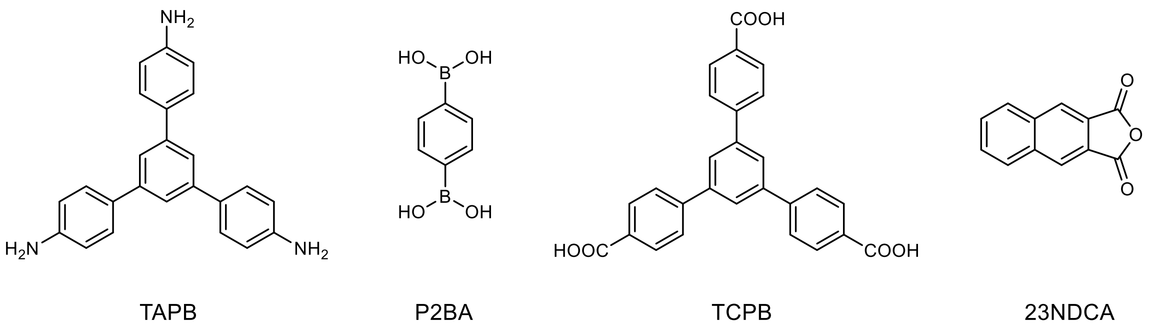

Supplementary Fig. 1 The chemical structures of TAPB, P2BA, TCPB, 23NDCA in the study.

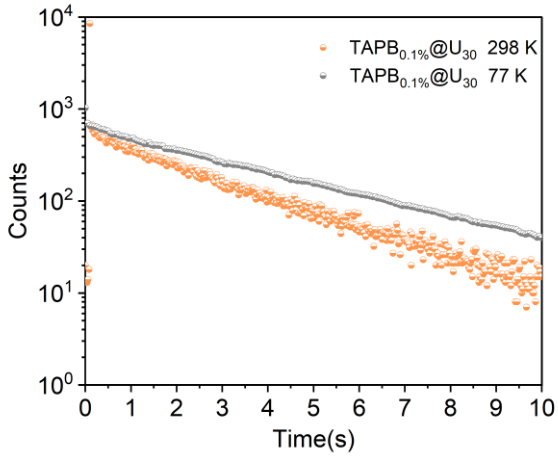

Supplementary Fig. 2 Phosphorescence decay curve of $TAPB_{0.1\%}@U_{30}$ at 298 and 77 K.

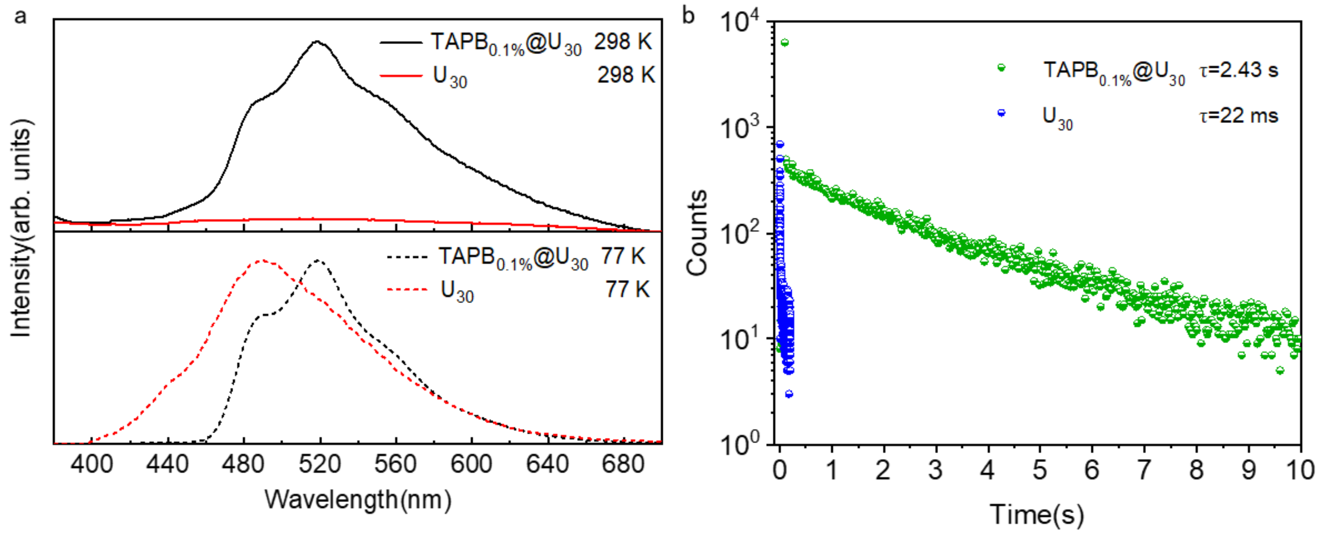

Supplementary Fig. 3 Photophysical properties of $TAPB_{0.1\%}@U_{30}$ and U_{30}. (a) Delayed photoluminescence (PL) spectra of $TAPB_{0.1\%}@U_{30}$ and U_{30} at 298 and 77 K (delay 1 ms); (b) Phosphorescence decay curve of $TAPB_{0.1\%}@U_{30}$ and U_{30} at 298 K.

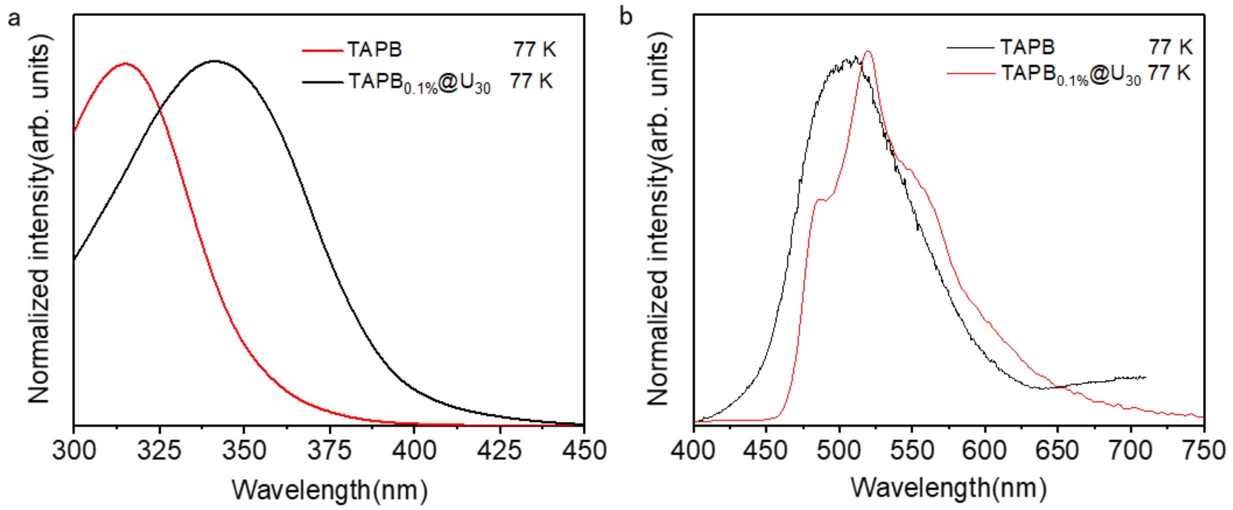

Supplementary Fig. 4 Photophysical properties of $TAPB_{0.1\%}@U_{30}$ and TAPB. (a) Normalized delayed PL spectra (delay 1 ms) and (b) normalized excitation spectra of TAPB in methanol and $TAPB_{0.1\%}@U_{30}$ solid at 77 K.

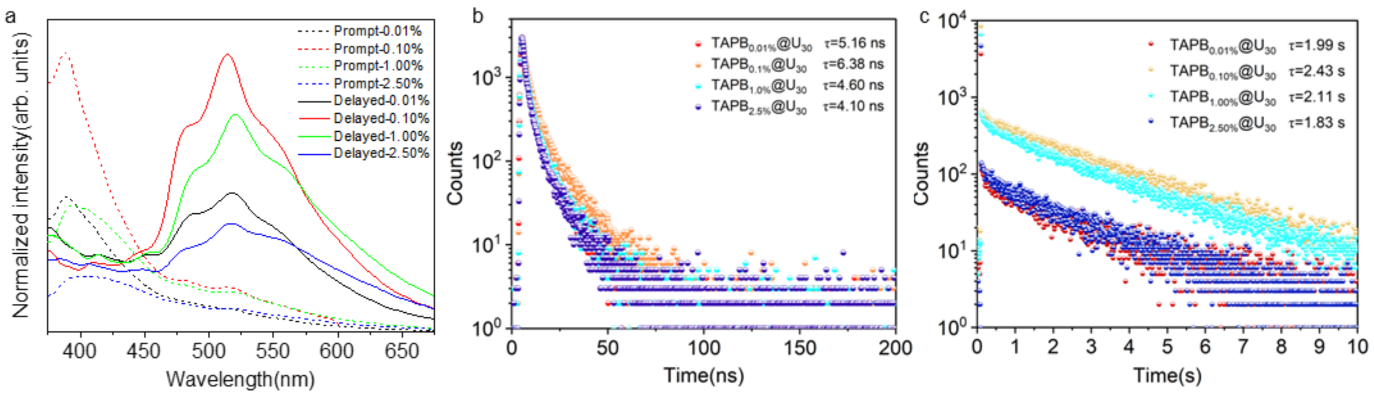

Supplementary Fig. 5 Photophysical properties of $TAPB_x@U_{30}$ (x = 0.01%, 0.10%, 1.00% and 2.50%). (a) Normalized prompt and delayed PL spectra (delay 1 ms) and (b) fluorescence decay curve, (c) phosphorescence decay curve of $TAPB_x@U_{30}$ (x = 0.01%, 0.10%, 1.00% and 2.50%) composites, excited at 365 nm and recorded at 519 nm, respectively.

Supplementary Table 1 Photophysical properties of $TAPB_x@U_{30}$ (x = 0.01%, 0.10%, 1.00% and 2.50%) composites at the room temperature, excited at 365 nm.

$TAPB_x@U_{30}$	λ_F [nm]	λ_{ph} [nm]	τ_F [ns]	τ_{Ph} [s]	Φ_F [%]	Φ_{Ph} [%]	K_rF (s^{-1})	$K_{nr}F$ (s^{-1})	K_{isc} (s^{-1})	K_rP (s^{-1})	$K_{nr}P$ (s^{-1})
**0.01%**	390	519	5.16	1.99	21.44	7.58	4.15×10^7	1.38×10^6	1.47×10^7	0.038	0.464
**0.10%**	391	519	6.38	2.43	10.13	5.37	1.59×10^7	1.32×10^6	8.42×10^6	0.022	0.389
**1.00%**	408	519	4.6	2.11	3.92	2.61	8.52×10^6	2.03×10^6	5.68×10^6	0.012	0.462
**2.50%**	428	523	4.1	1.83	1.92	1.96	4.68×10^6	2.34×10^6	4.79×10^6	0.011	0.536

λ_F: fluorescence emission; λ_{Ph}: phosphorescence emission; τ_F: fluorescence lifetime; τ_{Ph}: phosphorescence lifetime; Φ_F: fluorescence quantum yield; Φ_{Ph}: phosphorescence quantum yield; **K_rF**: radiative decay rate of fluorescence; **$K_{nr}F$**: nonradiative decay rate of fluorescence; **K_{isc}**: The intersystem crossing rate constant; **K_rP**: radiative decay rate of phosphorescence; **$K_{nr}P$**: nonradiative decay rate of phosphorescence; $K_rF=\Phi_F/\tau_F$; $K_{nr}F=(1-\Phi_F-\Phi_{ph})/\tau_F$; $K_{isc}=\Phi_{ph}/\tau_F$; $K_rP=\Phi_{Ph}/\tau_{Ph}$; $K_{nr}P=(1-\Phi_{Ph})/\tau_{Ph}$

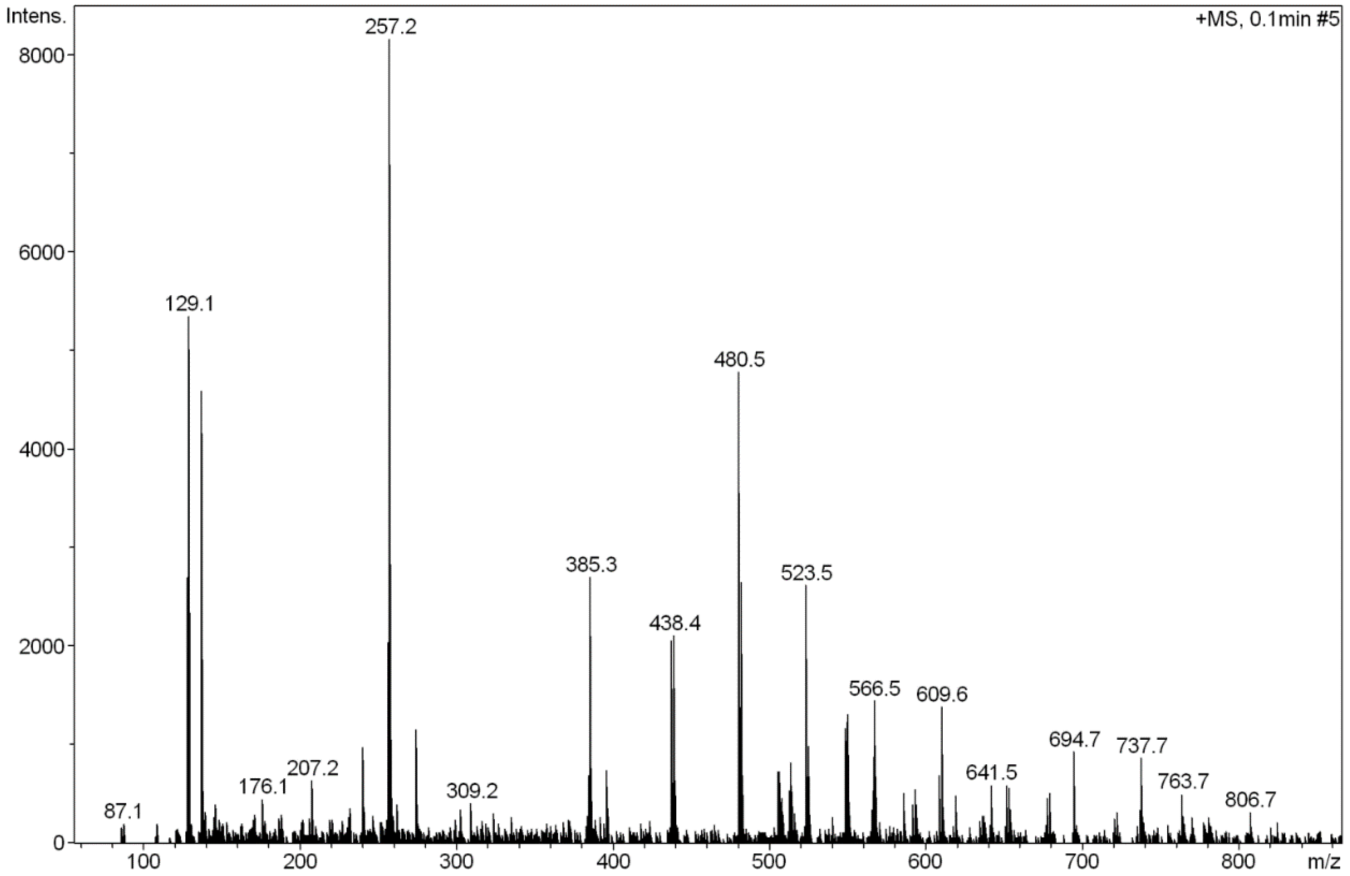

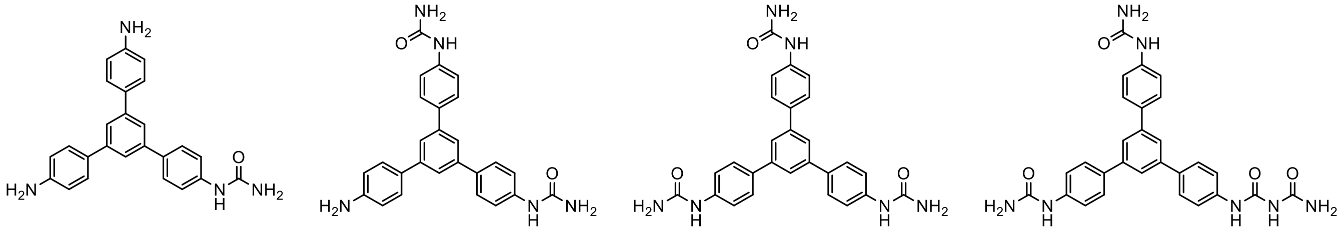

m/z=394.5 TAPB-1 | m/z=438.4 TAPB-2 | m/z=480.5 TAPB-3 | m/z=523.5 TAPB-4

Supplementary Fig. 6 High resolution mass spectrum and probable molecular units corresponding to the major peaks in mass spectrum of $TAPB_{1.0\%}@U_{30}$.

Supplementary Discussion 1

As shown in Fig. 3c, the characteristic peak attributed to urea at 22.2° could be clearly observed in $TAPB_{0.1\%}@U_{10}$, while the typical peaks of biuret appeared at 18.4°, 21.0°, 24.7°, 26.2°, and 31.5°. With the extension of pyrolysis time, the characteristic peaks of urea in $TAPB_{0.1\%}@U_{15}$ and $TAPB_{0.1\%}@U_{20}$ gradually disappeared, and the characteristic peaks of biuret gradually increased. However, the characteristic peaks of biuret significantly weakened in $TAPB_{0.1\%}@U_{25}$, and the peak at 28° attributed to ammelide rapidly increased. The XRD spectra of $TAPB_{0.1\%}@U_{30}$ and $TAPB_{0.1\%}@U_{35}$ almost completely showed the characteristic peaks of ammelide. These results indicated that the urea pyrolysis conversion pathway involved urea-biuret-ammelide (Fig. 3d).

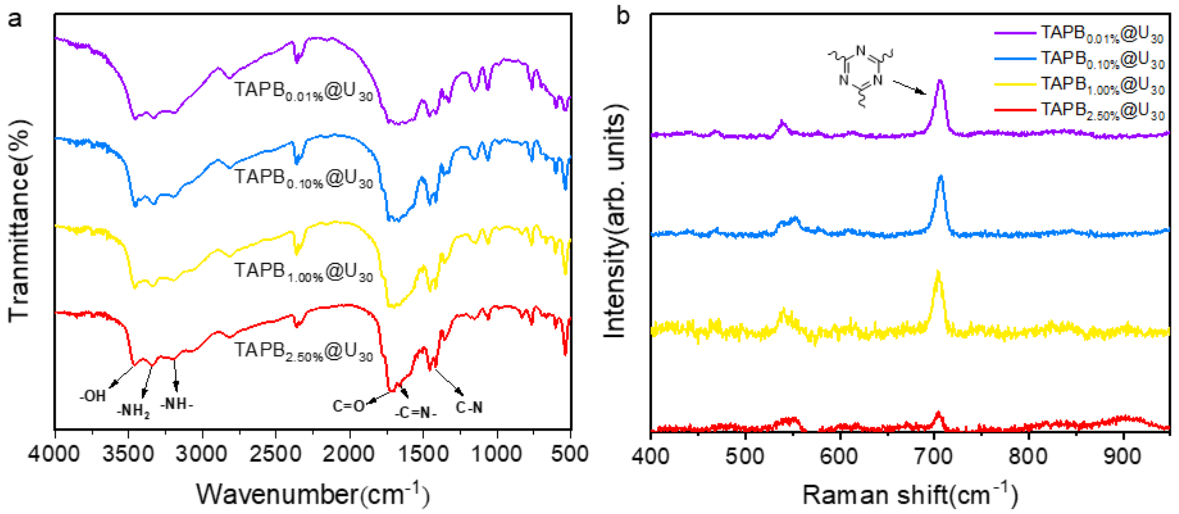

Supplementary Fig. 7 (a) Fourier transform infrared (FTIR) spectra and (b) Raman spectra of $TAPB_x@U_{30}$ (x = 0.01%, 0.10%, 1.00% and 2.50%) composites.

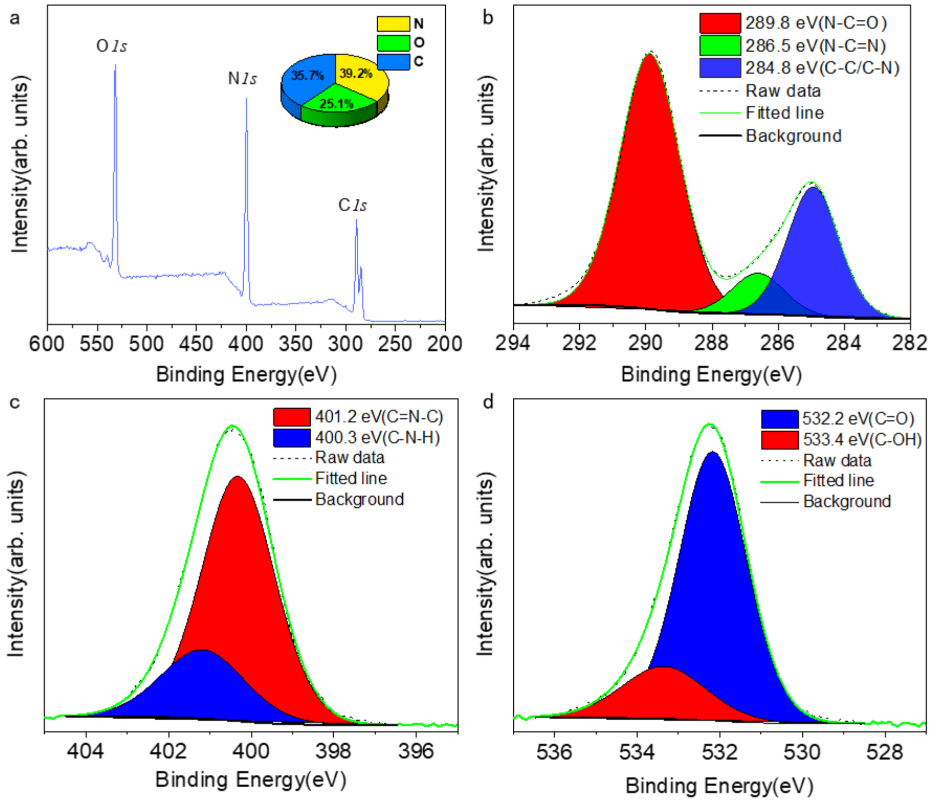

Supplementary Fig. 8 (a) X-ray photoelectron spectra (XPS) survey scan and (b) C*1s*, (c) N*1s*, (d) O*1s* HRXPS spectra of $TAPB_{0.1\%}@U_{30}$.

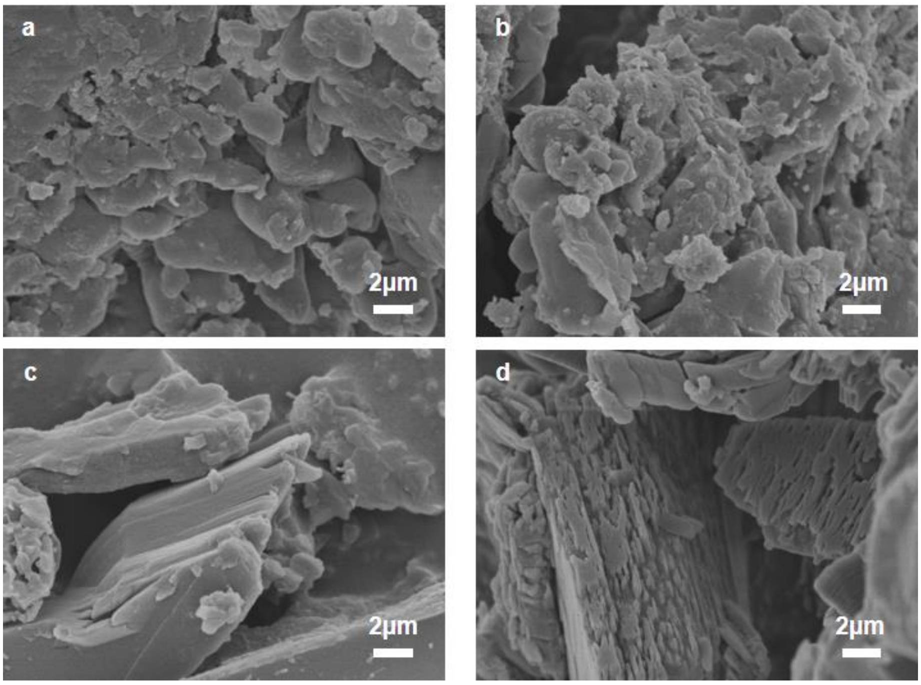

Supplementary Fig. 9 Scanning Electron Microscope (SEM) images of (a) (b) $TAPB_{0.1\%}@U_{10}$ and (c) (d) $TAPB_{0.1\%}@U_{30}$.

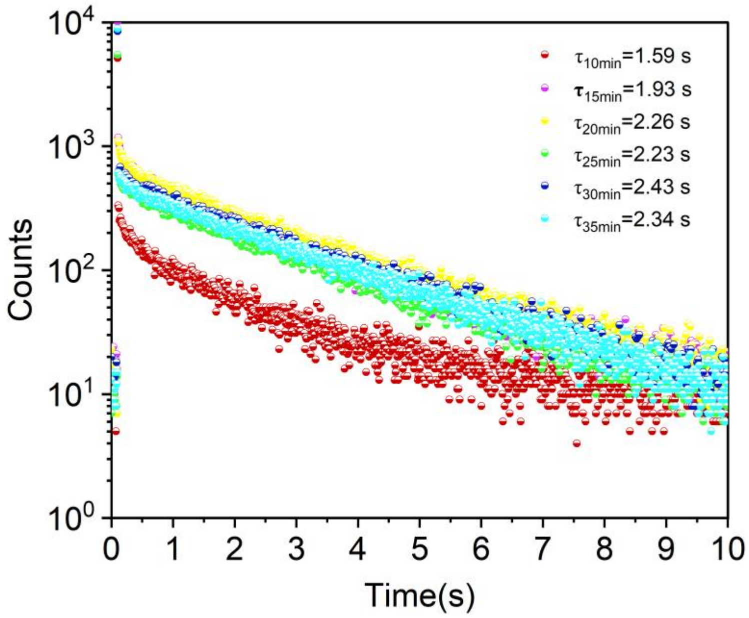

Supplementary Fig. 10 Phosphorescence decay curves of $TAPB_{0.1\%}@U_t$ (t=10, 15, 20, 25, 30, 35 min), excited at 365 nm and recorded at 519 nm, respectively.

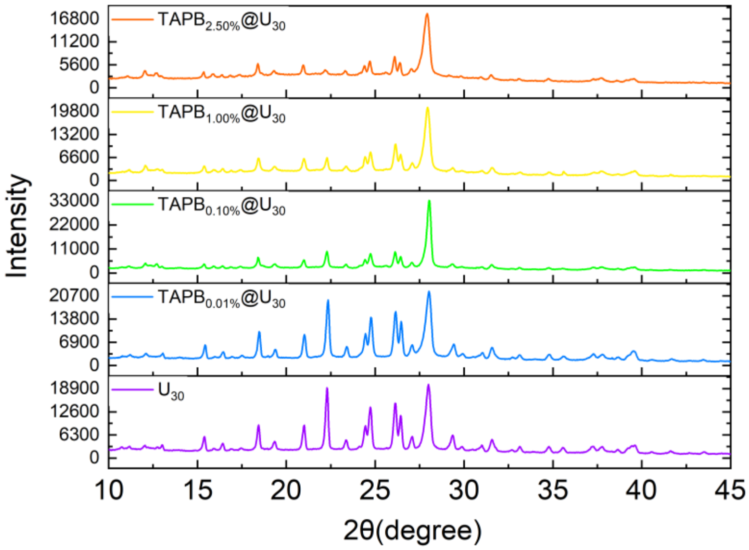

Supplementary Fig. 11 PXRD patterns of $TAPB_x@U_{30}$ (x = 0.01%, 0.10%, 1.00% and 2.50%) composites and urea pyrolyzed at 220 °C for 30 min.

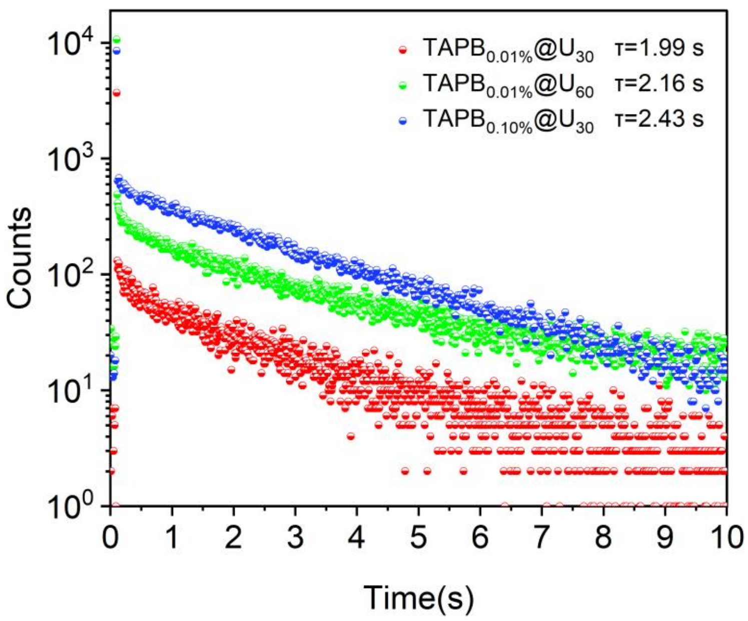

Supplementary Fig. 12 Phosphorescence decay curve of $TAPB_{0.01\%}@U_{30}$, $TAPB_{0.01\%}@U_{30}$ and $TAPB_{0.1\%}@U_{30}$.

Supplementary Discussion 2

As shown in Supplementary Fig. 11, some biurets still existed in $TAPB_{0.01\%}@U_{30}$, resulting in less satisfactory phosphorescent performance. $TAPB_{0.01\%}@U_{60}$ prepared by extending the pyrolysis time had the same matrix composition as $TAPB_{0.1\%}@U_{30}$, but its phosphorescence lifetime was still short due to inefficient energy transfer caused by lower content of guest molecules (Supplementary Fig. 12). Furthermore, poor crystallinity of the material caused by high content of guest molecules might be the main reason for the decrease in the RTP performance of $TAPB_{2.5\%}@U_{30}$ (Supplementary Fig. 11).

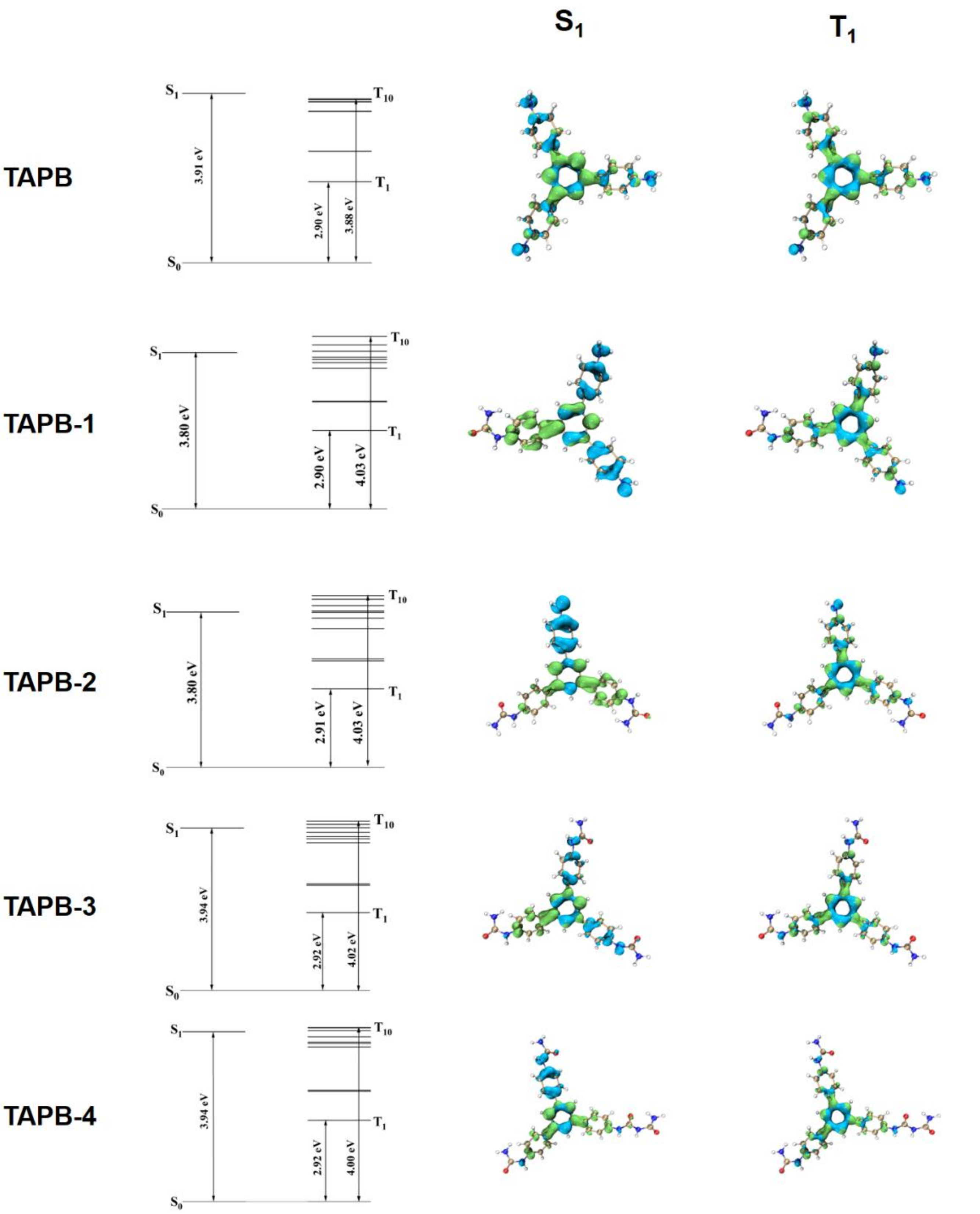

Supplementary Fig. 13 The energy level diagrams, electron−hole isosurface maps of the $S_0 \rightarrow S_1$, $S_0 \rightarrow T_1$ transition of the TAPB and TAPB-N compounds. Blue and green isosurfaces correspond to hole and electron distributions, respectively[1-4].

Supplementary Table 2 Quantitative characterization of the distribution of holes and electrons

	TAPB		TAPB-1		TAPB-2		TAPB-3		TAPB-4	
	$S_0 \to S_1$	$S_0 \to T_1$	$S_0 \to S_1$	$S_0 \to T_1$	$S_0 \to S_1$	$S_0 \to T_1$	$S_0 \to S_1$	$S_0 \to T_1$	$S_0 \to S_1$	$S_0 \to T_1$
**D-index**	0.343	0.029	2.807	0.181	3.779	0.209	1.540	0.070	1.818	0.054
**t-index**	-2.290	-2.512	0.366	-2.461	1.278	-2.477	-1.153	-2.679	-0.916	-2.655

D-index: measure of the distance between the hole and the electron center of mass; **t-index:** measure the degree of separation of holes and electrons.

Supplementary Table 3 Spin-orbit coupling (SOC) constants and singlet-triplet splitting energies (△E_{ST}) between the S_0/S_1 and involved T_n states of TAPB and TAPB-N compounds calculated in the gas phase.

	**TAPB**		**TAPB-1**		**TAPB-2**		**TAPB-3**		**TAPB-4**	
	**SOC**	**△E_{ST}**	**SOC**	**△E_{ST}**	**SOC**	**△E_{ST}**	**SOC**	**△E_{ST}**	**SOC**	**△E_{ST}**
**$S_0 \to T_1$**	0.30	2.903	0.34	2.895	0.37	2.905	0.36	2.921	0.34	2.92
**$S_1 \to T_1$**	0.27	1.010	0.30	0.901	0.29	0.897	0.35	1.021	0.39	1.022
**$S_1 \to T_2$**	0.03	0.664	0.14	0.569	0.35	0.568	0.20	0.686	0.17	0.691
**$S_1 \to T_3$**	0.02	0.663	0.30	0.552	0.05	0.540	0.09	0.669	0.17	0.671
**$S_1 \to T_4$**	0.32	0.244	0.03	0.186	0.19	0.196	0.08	0.170	0.05	0.170
**$S_1 \to T_5$**	0.18	0.239	0.58	0.127	0.60	0.090	0.18	0.116	0.37	0.095
**$S_1 \to T_6$**	0.16	0.238	0.30	0.085	0.13	-0.025	0.28	0.088	0.26	0.093
**$S_1 \to T_7$**	0.08	0.079	0.21	0.034	0.32	-0.053	0.40	0.066	0.32	0.057
**$S_1 \to T_8$**	0.16	0.075	0.26	-0.067	0.27	-0.080	0.09	-0.014	0.14	-0.021
**$S_1 \to T_9$**	0.22	0.057	0.17	-0.078	0.19	-0.154	0.56	-0.037	0.52	-0.033
**$S_1 \to T_{10}$**	0.30	0.038	0.02	-0.234	0.14	-0.226	0.26	-0.052	0.37	-0.078

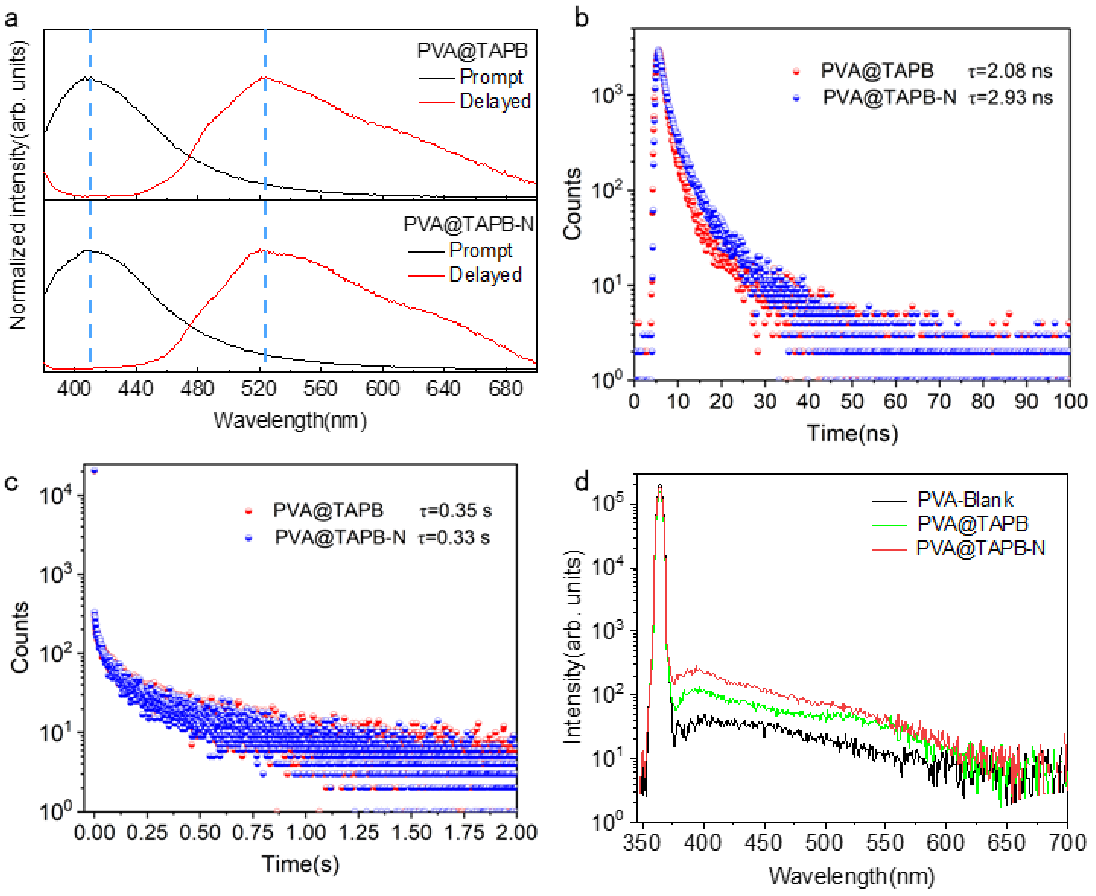

Supplementary Fig. 14 Photophysical properties of PVA@TAPB and PVA@TAPB-N. (a) Normalized prompt, delayed PL spectra (delay 1 ms), (b) fluorescence decay curve, (c) phosphorescence decay curve, (d) Total photoluminescence quantum yield curve of PVA@TAPB and PVA@TAPB-N.

Supplementary Table 4 Photophysical properties of TAPB and TAPB-N in PVA films under ambient conditions.

	λ_F [nm]	λ_{ph} [nm]	τ_F [ns]	τ_{Ph} [s]	Φ_F [%]	Φ_{Ph} [%]	K_rF (s^{-1})	$K_{nr}F$ (s^{-1})	K_{isc} (s^{-1})	K_rP (s^{-1})	$K_{nr}P$ (s^{-1})
**TAPB**	409	524	2.08	0.36	1.85	1.33	8.89×10^6	4.65×10^6	6.39×10^6	0.037	2.748
**TAPB-N**	411	523	2.93	0.33	10.18	4.87	3.47×10^7	2.90×10^6	1.66×10^7	0.148	2.891

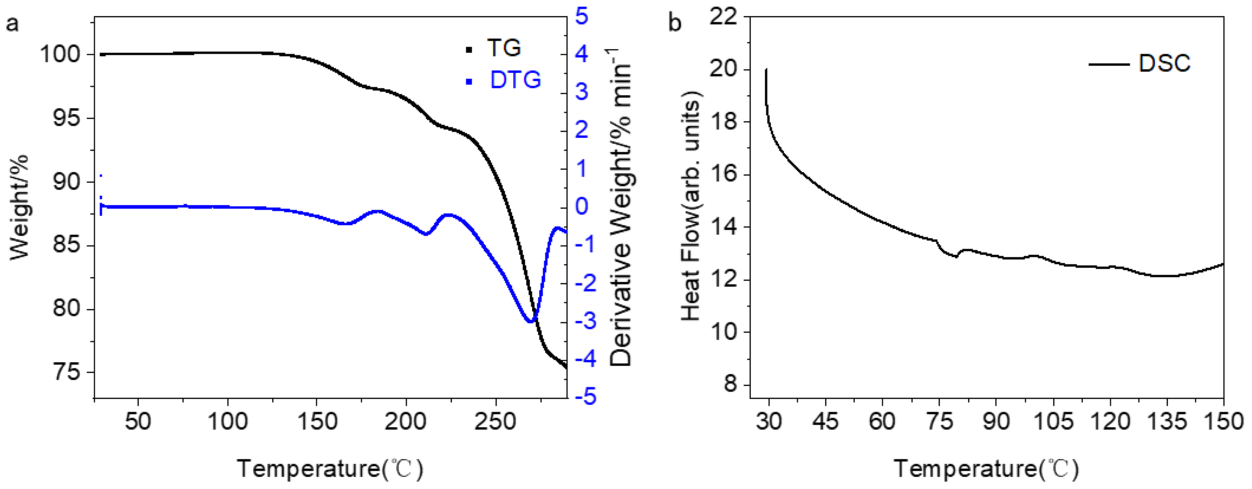

Supplementary Fig. 15 (a) Thermosgravimetric analysis (TGA) and derivative thermogravimetry (DTG) curves of $TAPB_{0.1\%}@U_{30}$; (b) differential scanning calorimetry (DSC) curve of $TAPB_{0.1\%}@U_{30}$.

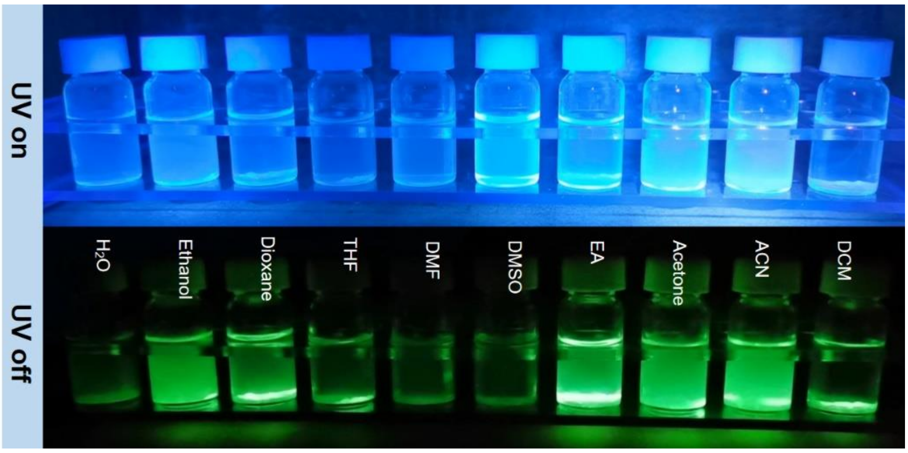

Supplementary Fig. 16 Luminescence photographs of $TAPB_{0.1\%}@U_{30}$ in different solvents under and after UV irradiation.

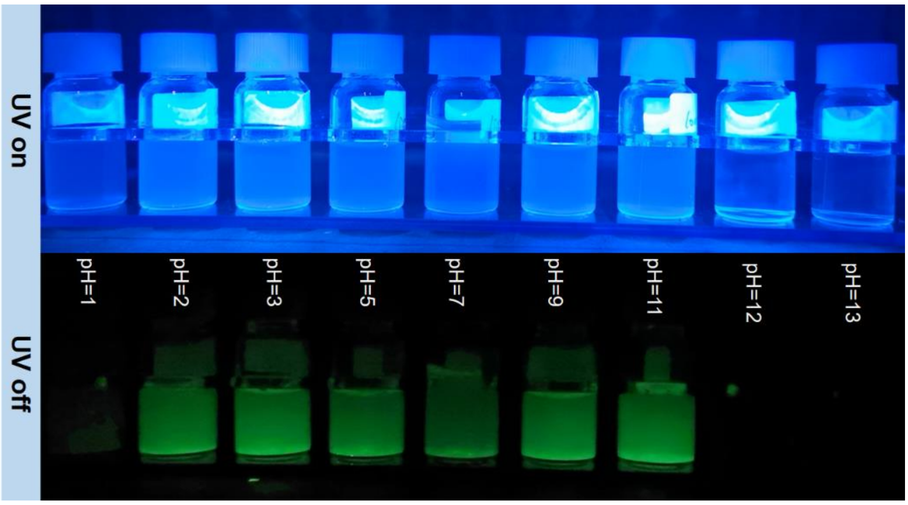

Supplementary Fig. 17 Luminescence photographs of $TAPB_{0.1\%}@U_{30}$ dispersed in water at different pH values under and after UV irradiation.

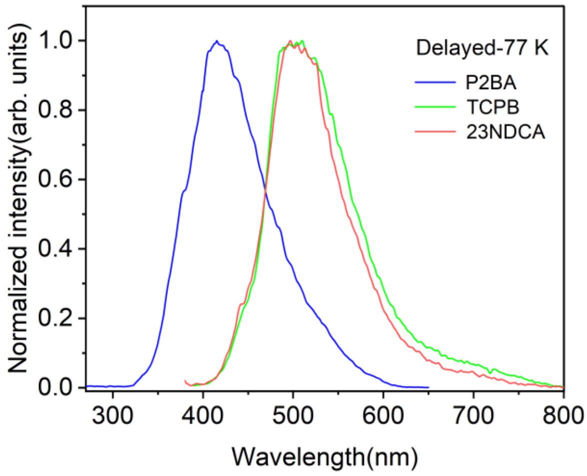

Supplementary Fig. 18 Delay emission spectra of P2BA, TCPB and 23NDCA solution (1×10^{-5} M) at 77 K (delay 1 ms).

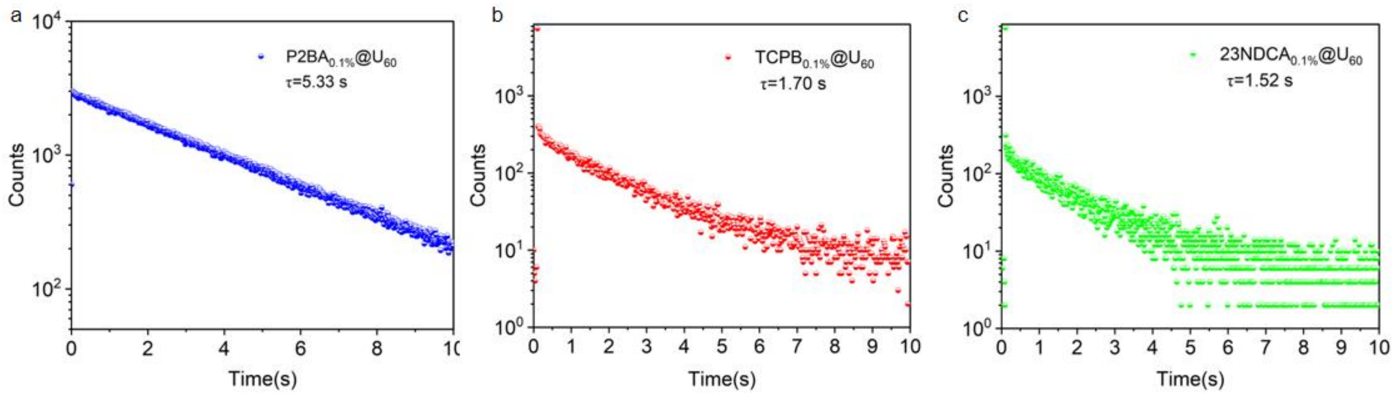

Supplementary Fig. 19 Phosphorescence decay curve of (a) $P2BA_{0.1\%}@U_{60}$; (b) $TCPB_{0.1\%}@U_{60}$ and (c) $23NDCA_{0.1\%}@U_{60}$.

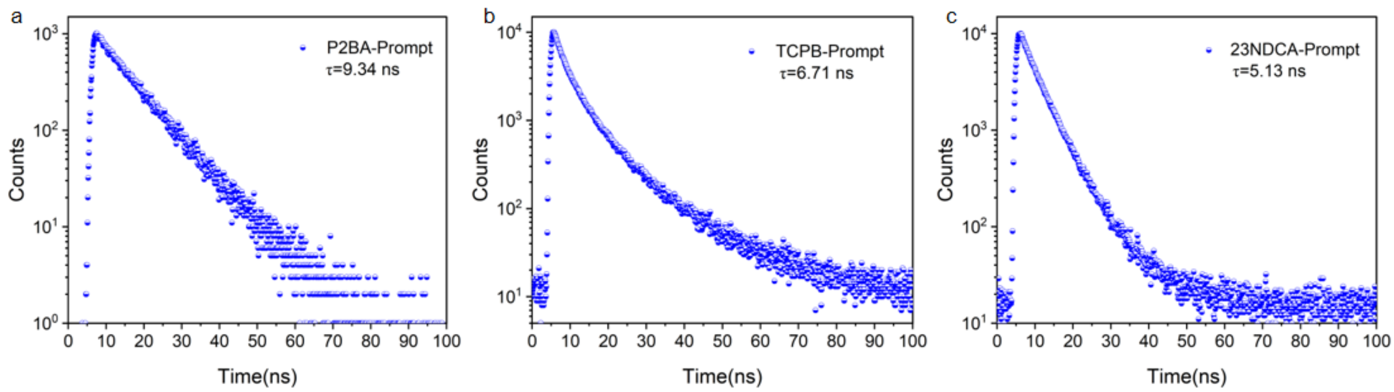

Supplementary Fig. 20 Fluorescence decay curve of (a) $P2BA_{0.1\%}@U_{60}$; (b) $TCPB_{0.1\%}@U_{60}$ and (c) $23NDCA_{0.1\%}@U_{60}$.

Supplementary Table 5 Photophysical properties of P2BA$_{0.1\%}$@U$_{60}$, TCPB$_{0.1\%}$@U$_{60}$ and 23NDCA$_{0.1\%}$@U$_{60}$.under ambient conditions.

	λ_F [nm]	λ_{ph} [nm]	τ_F [ns]	τ_{Ph} [s]	Φ_F [%]	Φ_{Ph} [%]	K_rF (s^{-1})	$K_{nr}F$ (s^{-1})	K_{isc} (s^{-1})	K_rP (s^{-1})	$K_{nr}P$ (s^{-1})
**P2BA**	328	417	9.34	5.33	3.30	10.82	3.53×10^6	9.19×10^5	1.16×10^7	0.020	0.167
**TCPB**	424	540	6.71	1.70	26.37	10.26	3.93×10^7	9.44×10^5	1.53×10^7	0.060	0.528
**23NDCA**	426	525	5.13	1.52	11.60	5.88	2.26×10^7	1.61×10^6	1.15×10^7	0.039	0.619

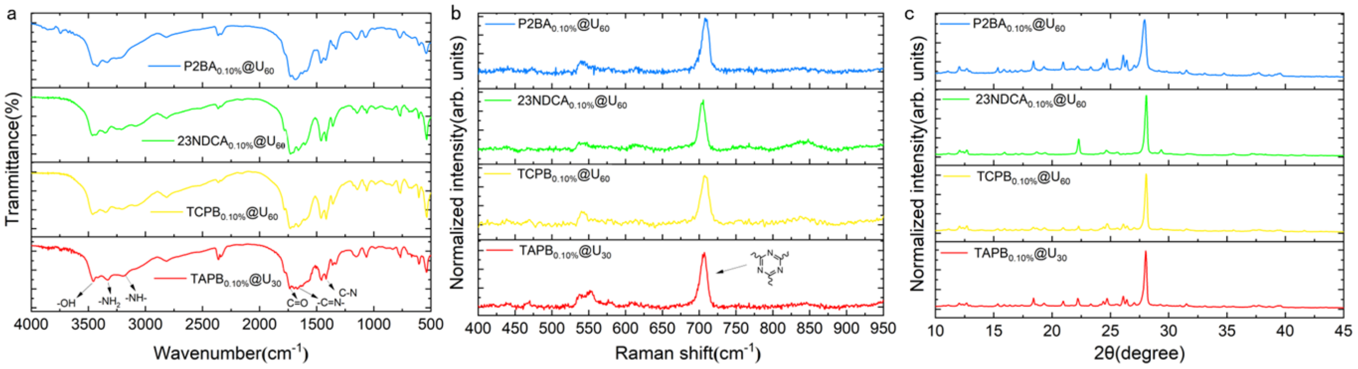

Supplementary Fig. 21 (a) FTIR spectra, (b) Raman spectra and (c) PXRD spectra of P2BA$_{0.1\%}$@U$_{60}$, TCPB$_{0.1\%}$@U$_{60}$, 23NDCA$_{0.1\%}$@U$_{60}$ and TAPB$_{0.1\%}$@U$_{30}$.

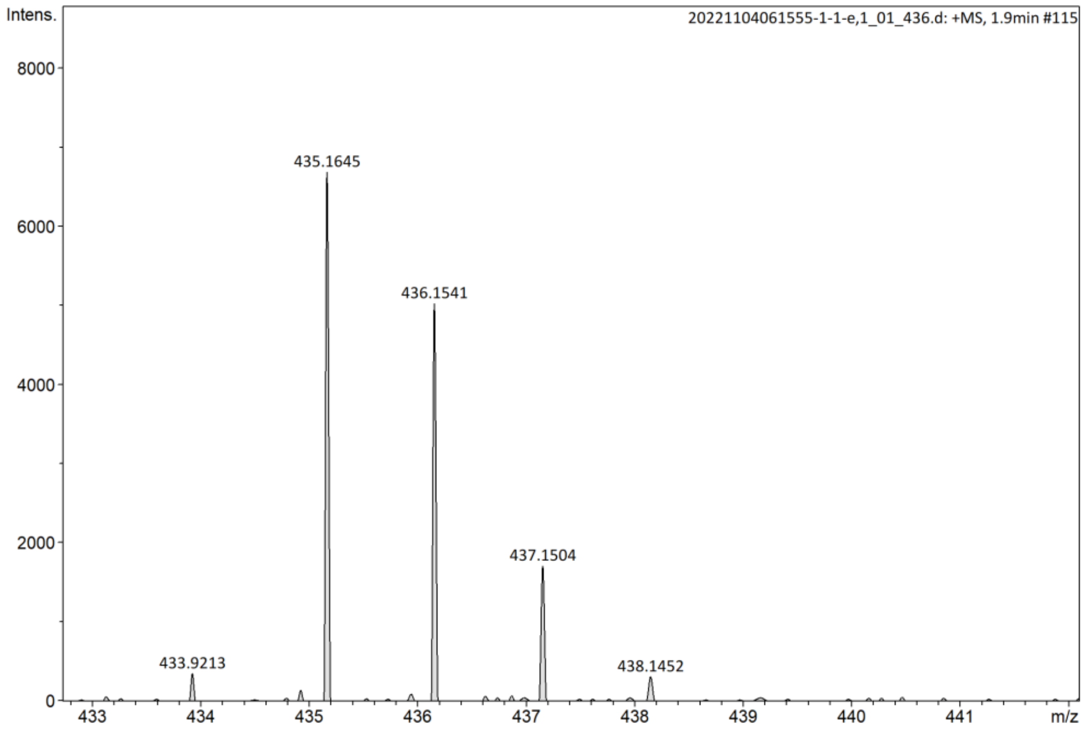

Supplementary Fig. 22 High resolution mass spectrum of TCPB$_{1.0\%}$@U$_{30}$.

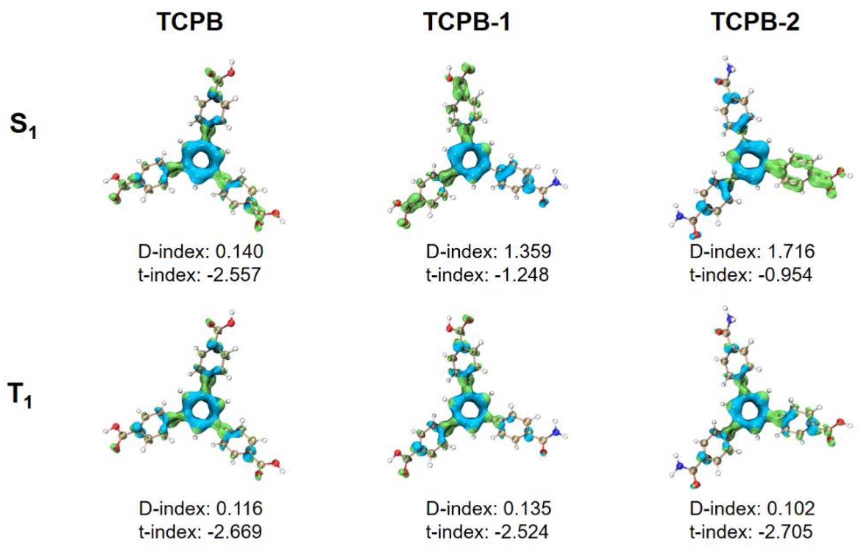

Supplementary Fig. 23 Electron–hole isosurface maps of the $S_0 \rightarrow S_1$, $S_0 \rightarrow T_1$ transition of the TCPB, TCPB-1 and TCPB-2. Blue and green isosurfaces correspond to hole and electron distributions.

Supplementary Table 6 Singlet-triplet splitting energies ($\triangle E_{ST}$) and spin-orbit coupling (SOC) constants between the S_0/S_1 and involved T_n states of TCPB and TCPB-N calculated in the gas phase.

	**TCPB**		**TCPB-1**		**TCPB-2**	
	**$\triangle E_{ST}$**	**SOC**	**$\triangle E_{ST}$**	**SOC**	**$\triangle E_{ST}$**	**SOC**
**$S_0 \rightarrow T_1$**	2.883	0.60	2.890	1.76	2.900	1.38
**$S_1 \rightarrow T_1$**	1.062	0.74	0.989	0.69	0.979	0.71
**$S_1 \rightarrow T_2$**	0.749	0.11	0.688	0.19	0.665	0.32
**$S_1 \rightarrow T_3$**	0.742	0.22	0.640	0.31	0.627	0.10
**$S_1 \rightarrow T_4$**	0.149	0.18	0.095	0.24	0.092	0.17
**$S_1 \rightarrow T_5$**	0.135	0.23	0.083	0.27	0.080	0.30
**$S_1 \rightarrow T_6$**	0.122	0.11	0.069	0.28	0.028	0.33
**$S_1 \rightarrow T_7$**	0.078	0.02	-0.051	0.19	-0.056	0.05
**$S_1 \rightarrow T_8$**	-0.063	0.27	-0.115	0.33	-0.108	0.50
**$S_1 \rightarrow T_9$**	-0.064	0.32	-0.126	0.30	-0.125	0.37
**$S_1 \rightarrow T_{10}$**	-0.066	0.26	-0.129	0.29	-0.129	0.52
**$S_1 \rightarrow T_{11}$**	/	/	-0.293	0.34	-0.291	0.52

Supplementary Discussion 3

As shown in Supplementary Fig. 22, the derivatives of TCPB were successfully generated during the co-pyrolysis of TCPB and urea. To evaluate similar effect of the derivative to TAPB-N in promoting ISC, hole-electron analysis was performed to describe the electron excitation characteristics of TCPB and its derivatives. As shown in Supplementary Fig. 23, the distribution of excited-state electron density of S_1 changed significantly due to the introduction of the amino group that served as an electron-donating moiety. Unlike carbonyl group, the introduction of amino group increased the electron cloud density near the amino region, further enhancing the separation of electrons and holes throughout the derivative molecule. Further calculations have also confirmed the important contribution of this transition to increasing the SOC constant and promoting the ISC process. Compared to TCPB, the SOC constant of derivatives significantly increased, which could provide more channels for ISC (Supplementary Table 6).

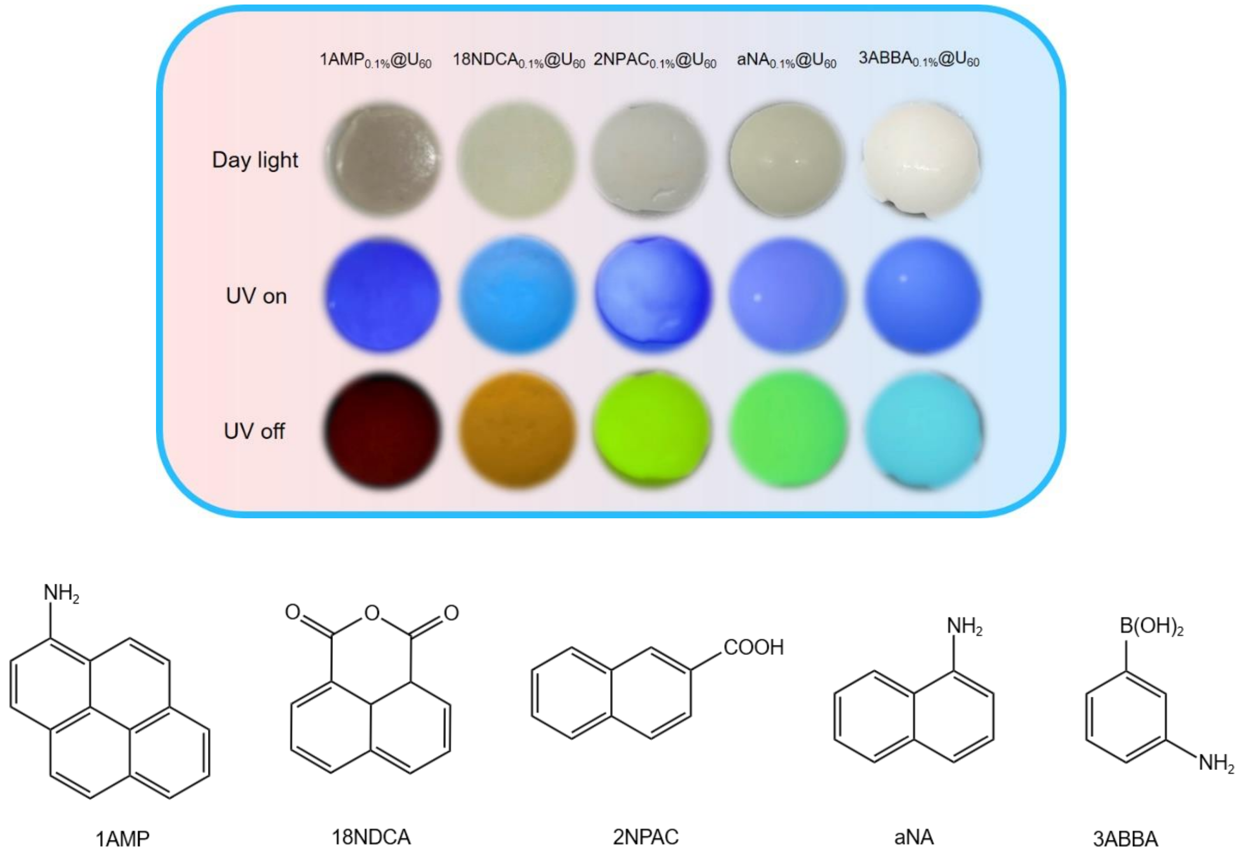

Supplementary Fig. 24 Photographs of polychromatic RTP composites before and after turning off 365 nm UV light, and the corresponding guest molecules of polychromatic RTP composites.

Supplementary Table 7 Some photophysical properties of polychromatic RTP composites.

**$XXX_{0.1\%}$@U_{60}**	**λ_F(nm)**	**λ_{Ph1}(nm)**	**τ_{Ph1}(s)**	**λ_{Ph2}(nm)**	**τ_{Ph2}(ms)**	**PLQY(%)**
**3ABBA**	409	454	1.13			70.38
**aNA**	392	490	1.48	520	1.52	14.63
**2NPAC**	417	540	0.96	593	0.93	59.50
**18NDCA**	478	567	0.29	607	0.34	11.94
**1AMP**	409	621	0.17	671	0.17	7.60

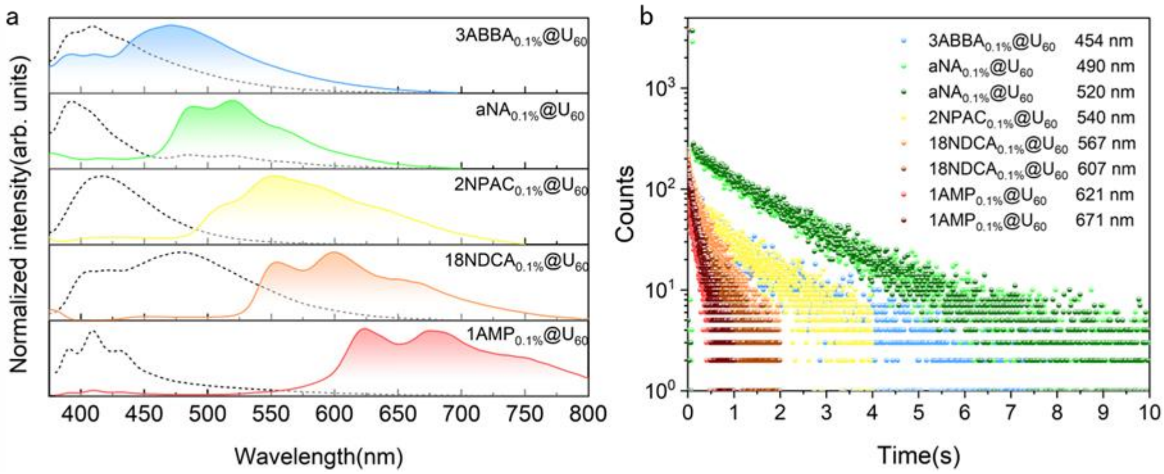

Supplementary Fig. 25 (a) Normalized prompt, delayed PL spectra (delay 1 ms) and (b) phosphorescence decay curve of $3ABBA_{0.1\%}$@U_{60}, $aNA_{0.1\%}$@U_{60}, $2NPAC_{0.1\%}$@U_{60}, $18NDCA_{0.1\%}$@U_{60} and $1AMP_{0.1\%}$@U_{60}.

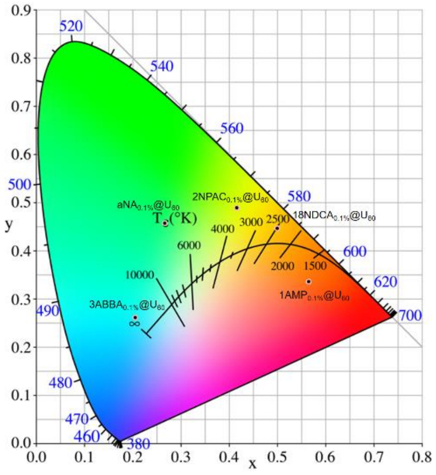

Supplementary Fig. 26 The CIE coordinates of $3ABBA_{0.1\%}$@U_{60}, $aNA_{0.1\%}$@U_{60}, $2NPAC_{0.1\%}$@U_{60}, $18NDCA_{0.1\%}$@U_{60} and $1AMP_{0.1\%}$@U_{60}.

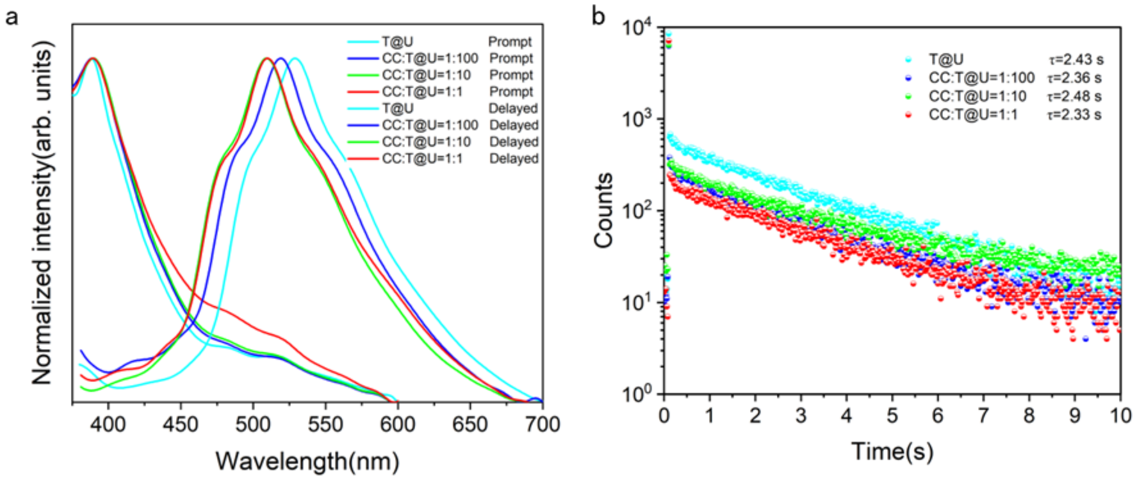

Supplementary Fig. 27 (a) Normalized prompt, delayed PL spectra (delay 1 ms) and (b) phosphorescence decay curve of $TAPB_{0.1\%}@U_{30}$ (T@U) with different content of choline chloride (CC).

Supplementary References

1 Lu, T. & Chen, F. Multiwfn: a multifunctional wavefunction analyzer. *J. Comput. Chem.* **33**, 580-592, doi:10.1002/jcc.22885 (2012).

2 Liu, Z., Lu, T. & Chen, Q. An sp-hybridized all-carboatomic ring, cyclo[18]carbon: Electronic structure, electronic spectrum, and optical nonlinearity. *Carbon* **165**, 461-467, doi:10.1016/j.carbon.2020.05.023 (2020).

3 Humphrey, W., Dalke, A. & Schulten, K. VMD: Visual Molecular Dynamics. *J. Mol. Graphics* **14**, 33-38 (1996).

4 Neese, F. Software update: the ORCA program system, version 4.0. *WIREs Computational Molecular Science* **8**, doi:10.1002/wcms.1327 (2017).
